# Perioperative functional imaging after extracranial carotid endarterectomy for the detection of cerebral hyperperfusion syndrome

**DOI:** 10.1007/s00423-022-02623-4

**Published:** 2022-07-29

**Authors:** Carola Marie Hoffmann-Wieker, U. Ronellenfitsch, F. Rengier, K. Otani, E. Stepina, D. Böckler

**Affiliations:** 1 Department of Vascular and Endovascular Surgery, University Hospital Heidelberg, Im Neuenheimer Feld 420, 69120 Heidelberg, Germany; 2 Department of Visceral, Vascular and Endocrine Surgery, University Hospital Halle (Saale), Halle (Saale), Germany; 3 Department of Diagnostic and Interventional Radiology, University Hospital Heidelberg, Heidelberg, Germany; 4 Siemens Healthcare K.K., Tokyo, Japan; 5 Siemens Healthcare GmbH, Forchheim, Germany

**Keywords:** Cerebral hyperperfusion syndrome, Cerebral blood volume, Carotid artery revascularization, Functional imaging

## Abstract

**Introduction:**

*SyngoDynaPBVNeuro®* is a tool to perform cerebral blood volume (CBV) measurements intraoperatively by functional imaging producing CT-like images. Aim of this prospective study was to analyze the clinical relevance and benefit of CBV measurement with regard to neurological complications like cerebral hyperfusion syndrome (CHS).

**Methods:**

Forty-five patients undergoing endarterectomy (CEA) of the internal carotid artery were included; functional imaging with CBV measurement was performed before and after CEA. To evaluate and analyze CBV, six regions of interest (ROI) were identified for all patients with an additional ROI in patients with symptomatic ICA stenosis and previous stroke. The primary endpoint of the study was a perioperative change in CBV measurements. Secondary outcomes were incidence of stroke, TIA, CHS, and perioperative morbidity and mortality.

**Results:**

Thirty-day stroke incidence and thirty-day mortality were 0%. Thirty-day morbidity was 6.7%. Two patients from the asymptomatic group suffered from transient neurological symptoms without signs of intracerebral infarction in CT or MR scan, meeting diagnostic criteria for CHS. In 83.3% of ROIs in these patients, an increase of blood volume was detected. Overall, 26.7% patients suffered from unilateral headache as expression of potential CHS. A total of 69.4% of ROIs in patients with postoperative unilateral headache showed an increase when comparing pre- and postoperative CBV measurements.

**Conclusion:**

The results show that increased CBV measured by functional imaging is a possible surrogate marker of neurological complications like CHS after CEA. By using intraoperative CBV measurement, the risk of CHS can be estimated early and appropriate therapeutic measures can be applied.

## Introduction

Carotid endarterectomy (CEA) for internal carotid artery (ICA) stenosis is safe, effective, and represents, according to guidelines, the first choice treatment in symptomatic and selected asymptomatic patients with a relevant degree of stenosis [1–7]. CEA is one of the most frequently performed operations in vascular surgery. In high-volume centers, perioperative morbidity is low. Besides irreversible neurological complications like stroke or cerebral hemorrhage, the development of cerebral hyperperfusion syndrome (CHS) after carotid endarterectomy or carotid angioplasty is possible [8]. CHS was first defined by Sundt et al. in 1981 by analyzing cerebral blood flow measurements of over 1000 patients after CEA as increased cerebral blood flow (CBF) “to more than 200% of baseline flow” [9]. Risk factors for development of CHS are reduced cerebral vasoreactivity, contralateral ICA stenosis of ≥ 70%, post-procedural hypertension, and recent ipsilateral stroke. Baroreceptor dysfunction after carotid surgery can lead to impaired cerebral autoregulation, which in combination with increased CBF contributes to the development of CHS. There is no commonly agreed consensus on diagnostic criteria for CHS. Usually CHS is considered in patients suffering from severe unilateral headache, transient seizures, focal neurological deficits, migraine variants, and, rarely, intracerebral or subarachnoidal hemorrhage [8–10]. The latter is associated with high morbidity and mortality [9–16]. CHS occurs with an incidence of 0.2 to 18.9% in patients undergoing CEA. Because of the serious complications, early evaluation and diagnosis of CHS are important [17–21]. The condition is treated by early strict blood pressure management, which is crucial to avoid the severe sequelae described above.

Functional imaging techniques like *syngo DynaPBV Neuro®* measure cerebral blood volume (CBV) by characterizing the difference in contrast agent distribution in brain tissue using a C-arm flat detector angiographic system along with a multislice perfusion CT scan [22, 23]. In modern hybrid operation suites equipped with the possibility of rotational imaging producing CT-like images, CBV measurements can be performed intraoperatively. The aim of this study was to evaluate the utility of intraoperative CBV measurement for early detection of CHS, which is an underrecognized yet sometimes severe complication after carotid endarterectomy (CEA).

## Methods

### Inclusion criteria and study design

This prospective non-randomized study comprised patients undergoing CEA for carotid stenosis between January 2014 and January 2017. In order to enhance validity of CBV measurements, only patients with unilateral carotid stenosis were included. Patients with contralateral stenosis of > 50% and patients with acute or chronic renal failure and creatinine measurements > 1.2 mg/dl or glomerular filtration rate (GFR) < 40 ml/min, as well as patients with intolerance to contrast medium, were excluded. Demographic and clinical data including postoperative blood pressure and neurological status and follow-up data were collected prospectively. Blood pressure was documented preoperatively, at the end of the procedure, and 4, 24, 48, and 72 h postoperatively. The measurements preoperatively, at the end of the procedure and 4 h postoperatively, were done by invasive intraarterial measurement, while at 24, 48, and 72 h, blood pressure was measured with a noninvasive sphygmomanometer three times a day or more frequently in case of hypertension. Arterial hypertension was defined as systolic blood pressure ≥ 140 mmHg regarding to the European Guidelines for management of arterial hypertension [24].

Specific conditions like unilateral headache and neurological symptoms including stroke or transient ischemic attack (TIA) were documented at these time points, too. All patients were followed up by vascular surgeons in an outpatient clinic 6 weeks postoperatively, and then at least annually. Routine follow-up was done by duplex scan and, in selected cases with pathological duplex findings, CT-angiography. During follow-up visits, the patients were asked and examined for persistent neurological findings, e.g., persistent cranial nerve injury or persistent arterial hypertension. The study protocol was approved by the competent ethics committee (Medical Faculty of the University of Heidelberg, Germany, reference number: S-054/2014), and written informed consent was obtained from all patients.

### Functional imaging syngo DynaPBV Neuro®

CBV was measured in all patients by using a biplanar C-arm flat detector angiographic system (Artis zeego; Siemens Healthcare GmbH, Forchheim, Germany). In all patients, CBV measurements were performed in the operation room prior and post-surgery. The neuro parenchymal blood volume system (syngo NeuroPBV IR, Siemens Healthcare GmbH, Forchheim, Germany) was used. The protocol for functional imaging was the same protocol Struffert et al. published in 2010 [23]. Acquisition consisted of an initial rotation (mask run) followed by a second rotation after appropriate contrast medium injection (fill run). CBV is obtained by using a contrast medium injection protocol that results in a steady state of contrast medium in the brain parenchyma during acquisition of the fill run. The fill run was manually initiated when contrast filling of the transverse sinus was observed. Eighty milliliters of contrast medium (Imeron 300®, Bracco Imaging, Konstanz, Germany) was injected intravenously at a rate of 4 ml/s by using a power injector. Cerebral regions of interests (ROIs) were defined in size and brain positioning in reference to the study by Struffert et al. The following ROIs were used: (1) lateral basal ganglia, (2) frontal subcortical white matter, (3) occipital subcortical white matter, (4) thalamus, (5) corona radiata, and (6) internal capsule. All measurements were carried out by the principal investigator using identical ROIs in all patients. Instead of using mean CBV change or mean absolute CBV, a possible change from pre- to postprocedural CBV was assessed in a dichotomous way, where a change was defined as any increase from pre- to postprocedural CBV. In addition, three independent observers blinded to clinical outcomes assessed changes in CBV by analyzing the color-coded images provided by the *syngo DynaPBV Neuro®* software for all patients. They stated if there was an increase, decrease, or steady state in CBV.

### Surgical procedure

**Fig. 1:**
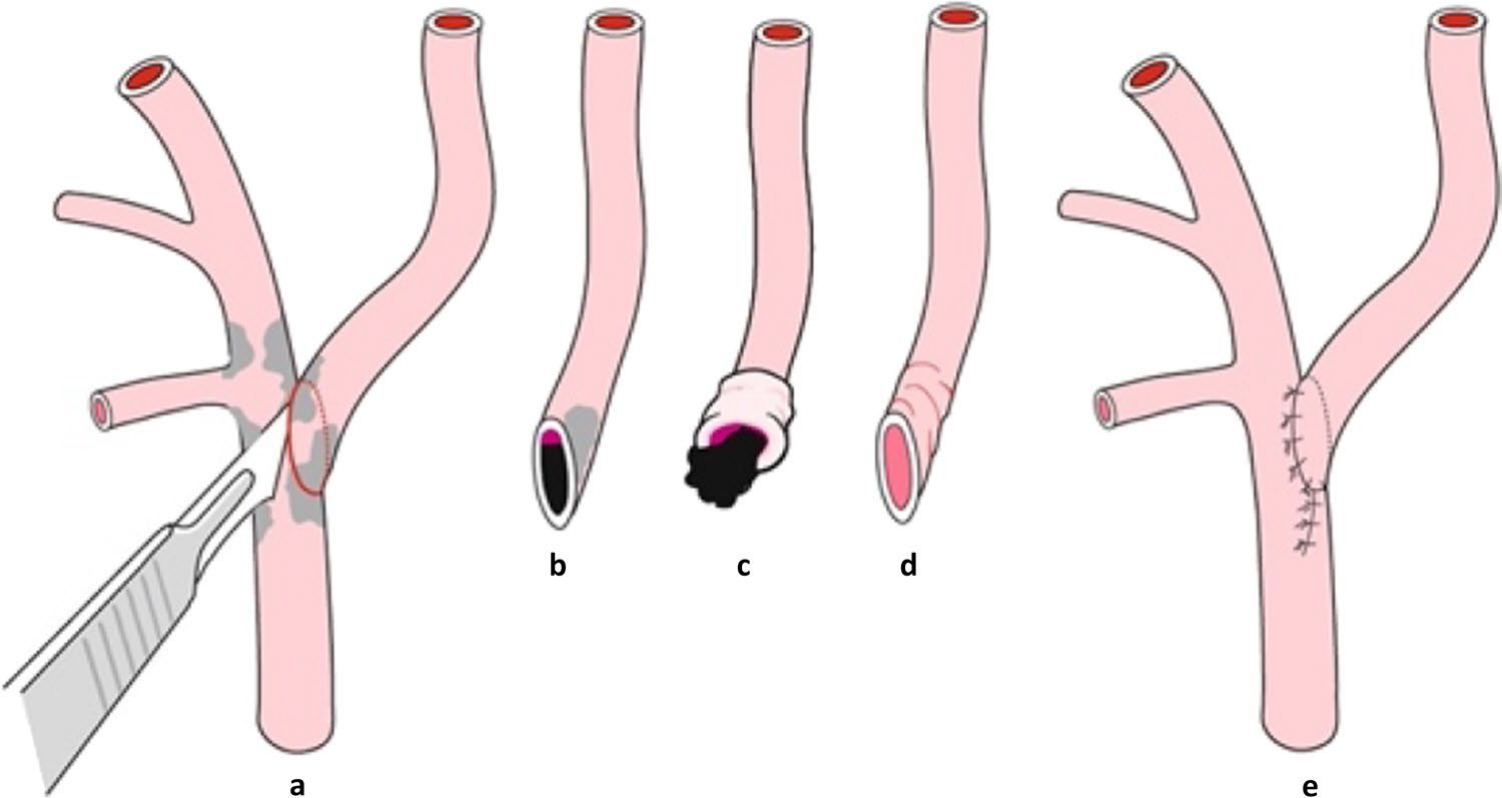
Technique of eversion endarterectomy (eCEA). **a** Dissection of ICA. **b**–**d** Eversion and endarterectomy of ICA. **e** Reinsertion of ICA. ICA: internal carotid artery

**Fig. 2:**
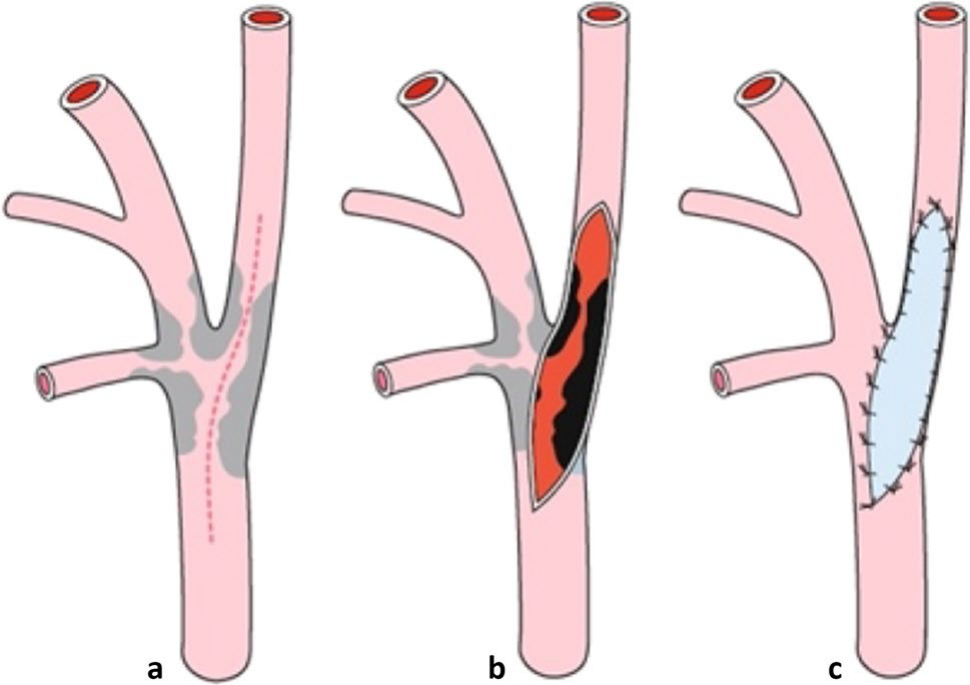
Technique of conventional endarterectomy with patchy plasty (cCEA). **a** Incision of ICA. **b** Endarterectomy of ICA. **c** Patch plasty of ICA. ICA: internal carotid artery

CEA was performed by using either institutionally preferred eversion endarterectomy (eCEA) or conventional endarterectomy with patch plasty (cCEA). Surgical techniques are shown in Figs. 1 and 2. eCEA was performed in patients with short length of stenosis and normally located carotid bifurcation. Depending on intraoperative anatomy or indication for primary or secondary shunt insertion, cCEA was performed. Primary shunt insertion from the common carotid artery to the ICA with the need for cCEA was performed in all patients with symptomatic ICA stenosis and recent intracranial infarction on cranial CT or MRT scan. A secondary shunt was inserted in all patients with a newly onset clinical neurological deficit (sensomotoric or cognitive function loss) upon clamping. Forty-three out of 45 patients (95.5%) underwent the operation in locoregional anesthesia; in two patients, conversion from locoregional to general anesthesia was necessary. All procedures were performed by or under direct supervision of a board-certified senior vascular surgeon. Before clamping, patients received 3000 IU heparin i.v. The operation was preferably conducted in cervical plexus anesthesia to allow continuous intraoperative assessment of the neurological status. Prior to surgery, all patients initiated a permanent antiplatelet therapy with acetylsalicylic acid (ASA) 100 mg/d or clopidogrel 75 mg/d. In patients with indication for anticoagulation therapy, the medication was changed prior to surgery to low molecular weight or unfractionated heparin. Besides CBV measurements as described above, all patients underwent intraoperative biplanar completion angiography (anterioposterior and 30° lateral position) of the extracranial reconstruction. Patients with symptomatic ICA stenosis additionally received biplanar intracranial angiography [7]. Postoperatively, a mean arterial blood pressure of 80–100 mmHg was tolerated in order to avoid excessive blood pressure lowering by antihypertensive medication in hypertensive patients accustomed to high blood pressure values.

### Study endpoints

The primary endpoint of the study was a perioperative change in CBV measurements as defined above. Secondary endpoints were periprocedural stroke, TIA, postoperative CHS, and perioperative mortality and morbidity including postoperative hypertension. Periprocedural stroke was defined as any new-onset neurological deficit within 30 days of the operation, lasting more than 24 h. A neurological impairment lasting less than 24 h was considered a TIA. The combination of ipsilateral headache on the CEA side and transient neurological symptoms not diagnosed as stroke or TIA by a board-certified neurologist was defined as CHS. Ipsilateral headache without neurological symptoms was considered a symptom suggestive of CHS.

### Statistical analysis

Continuous variables with normal distributions were presented as mean ± SD (standard deviation) or as median and interquartile range in case of non-normally distributed variables. The assumption of normal distribution was examined on quantile–quantile plots and tested by the Shapiro–Wilk test for each variable. Pre-operative and post-operative CBV values of each ROI in the hemisphere of the CEA were compared with the Wilcoxon matched-pairs signed-rank test. Pre- to post-operative CBV changes in the hemisphere of the CEA were compared with the Mann–Whitney *U* test (1) between patients with and without CHS, (2) between patients with and without arterial hypertension, and (3) between patients with symptomatic and asymptomatic ICA stenosis. The Bonferroni correction for multiple testing was not applied, as this study was considered exploratory.

The inter-observer agreement between three observers was assessed using Fleiss’ kappa coefficient and interpreted according to the scale proposed by Landis and Koch [25].

All analyses were performed with commercial software (Stata Statistical Software release 15, StataCorp LLC, College Station, TX, USA, and Microsoft Excel (2010), USA).

## Results

### Patient characteristics and procedural results

During the study period, 718 patients underwent carotid surgery at our institution. Out of these, 673 patients could not be included due to unavailability of the neuro parenchymal blood volume system, contralateral carotid stenosis > 50%, acute or chronic renal failure, or intolerance to contrast medium. Thus, the study included a total of 45 patients (median age 72, 66.6% male (*n* = 30/45)). Baseline characteristics and demographics of patients are shown in Table 1. Indication for carotid revascularization was an asymptomatic ICA stenosis of at least 70% in 25 patients (55.6%) and a symptomatic ICA stenosis of at least 50% in 20 patients (44.4%). The mean preoperative degree of stenosis was 80% in both groups. In the symptomatic ICA stenosis group, eCEA was performed in ten patients (50%), and cCEA with primary insertion of a temporary shunt in seven patients (35%). Two patients (10%) underwent cCEA without shunt insertion (no cerebral infarction detected on preoperative CT or MRT scan), and in one patient (5%), a secondary shunt insertion was needed because of onset of neurological symptoms upon clamping. In the asymptomatic ICA stenosis group, eCEA was done in 15 patients (60%) while eight patients (32%) underwent cCEA. There was no need for primary shunt insertion, but in one patient, secondary shunt insertion during cCEA was necessary because of onset of neurological symptoms upon clamping. One procedure (4%) consisted of ICA resection followed by carotid graft interposition.

**Table 1:** Baseline characteristics and demographics

	*n* = 45	Percentage (%)
Age (median)	72	
Gender (male)	*n* = 30	66.7
Cardiovascular risk factors		
Hypertension	*n* = 40	88.9
Dyslipidaemia	*n* = 43	95.5
Current smoking	*n* = 10	22.2
Coronary artery disease	*n* = 19	42.2
Previous myocardial infarction	*n* = 12	26.6
Diabetes mellitus	*n* = 10	22.2
Peripheral artery disease	*n* = 16	35.5
Symptomatic ICA stenosis	*n* = 20	44.4
Rankin scale		
0	*n* = 12	60
1	*n* = 5	25
2	*n* = 3	15

In 17.7% of procedures (8/45), there was a need for immediate surgical revision based on findings detected by intraoperative completion angiography. All revisions were successful as shown by repeat completion angiography. During a mean follow-up of 9 months (range 1–62 months), two patients (5.9%; *n* = 2/34) presented with intermittent hypertension requiring further antihypertensive treatment. During follow-up, there was no recurrent stenosis with a degree of stenosis > 50%. Patients’ survival during 6 weeks of follow-up was 97.8%.

### Primary endpoint

A total of 68.2% of ROIs in the hemisphere of the CEA side showed an increase when comparing pre- and postoperative CBV values (*p* < 0.001; 71.3% of ROIs in patients with asymptomatic stenosis and 65% of ROIs in patients with symptomatic stenosis); these results are shown in Table 2. Among patients who had suffered cerebral infarction, 87.5% showed an increase from pre- to postoperative CBV measurements in the ROI corresponding to the location of the infarction. At the contralateral (not operated) side, a CBV increase was observed in 61.1% of ROIs. The results of the comparison of change in pre-procedural to post-procedural CBV between patient groups are shown in Table 3. For patients with CHS compared to patients without CHS, there was no statistically significant difference in the percentage of ROIs with an increase in CBV (*p* = 0.24). The same was true for patients with postoperative hypertension compared to no postoperative hypertension (*p* = 0.4). There was however a significant difference between patients with asymptomatic compared to symptomatic ICA stenosis (*p* < 0.01). The mean preoperative degree of stenosis was 80% in both groups without differences in degree of stenosis.

**Table 2:** Comparison of pre-procedural and post-procedural CBV values in the hemisphere of the CEA side

ROI	Post-procedural increase	*p*-value (pre-procedural CBV vs post-procedural CBV)
1	Yes	< 0.001
2		0.05
3	Yes	0.03
4		0.35
5	Yes	< 0.001
6		0.06
All ROIs	Yes	< 0.001

**Table 3:** Comparison of change from pre-procedural to post-procedural CBV between patient groups

ROI	*p*-value (CHS vs no CHS)	*p*-value (arterial hypertension (AHT) vs no AHT)	*p*-value (symptomatic vs asymptomatic ICA stenosis)
1	0.53	0.65	0.62
2	0.79	0.39	0.33
3	0.54	0.72	0.06
4	0.23	0.69	0.15
5	0.31	0.52	0.39
6	0.25	0.98	0.58
All ROIs	0.24	0.40	0.01

The incidence of postoperative neurological events was 4.4% (*n* = 2/45). Two patients with asymptomatic ICA stenosis suffered from transient neurological symptoms. One of these patients was the only patient who needed secondary shunt insertion during cCEA because of onset of neurological symptoms upon clamping, upon which the neurological symptoms quickly subsided. Both patients had postoperative unilateral headache. In the first patient, new postoperative neurological symptoms with subtle motor impairment of the left hand occurred after a symptom-free interval. The cranial CT and CT-angiography were without pathological findings. The neurological symptoms remitted completely within a few hours. The second patient developed a hemiparesis of the contralateral upper extremity. Cranial CT and CT-angiography were without pathological findings. The patient was asymptomatic with completely remitted neurological symptoms at discharge. Both patients were diagnosed with CHS by a board-certified neurologist. In these patients, there was an increase in 83.3% of ROIs when comparing pre- and postoperative CBV measurements.

### Secondary endpoints

The overall perioperative complication rate was 6.7% (*n* = 3/45). Thirty-day mortality and stroke incidence were zero. The two patients with CHS were by definition counted as having a perioperative complication. One patient in the group with asymptomatic ICA stenosis suffered from hypoglossal nerve palsy, which was permanent.

Results of perioperative antihypertensive medication and CHS in the group of patients with asymptomatic ICA stenosis vs the group of patients with symptomatic ICA stenosis are shown in Table 4. None of the patients in the group with symptomatic ICA stenosis had postoperative complications. One patient with preoperative stroke had intraoperative neurological symptoms with suspicion of CHS not upon clamping but only after shunt insertion. In 66.6% of ROIs, there was significantly increased CBV. After the operation, the patient was asymptomatic and had no headache. Therefore, he was by definition not considered as having CHS. He was discharged from hospital after an uneventful postoperative course.

**Table 4:** Perioperative antihypertensive medication and CHS: asymptomatic ICA stenosis group vs symptomatic ICA stenosis group

	Asymptomatic ICA stenosis (*n* = 25) (*n*/%)	Symptomatic ICA stenosis (*n* = 20) (*n*/%)
Postoperative additional antihypertensive medication	15/60	15/75
Postoperative unilateral headache as possible clinical sign of CHS	8/32	4/20
Postoperative neurological symptoms diagnosed as CHS	2/8	0/0

*CHS* cerebral hyperperfusion syndrome

The median postoperative blood pressure was 140 mmHg (range 80–200) in patients with asymptomatic ICA stenosis and 150 mmHg (range 100–220) in patients with symptomatic ICA stenosis. A total of 66.7% (*n* = 30/45) of patients needed acute postoperative antihypertensive medication. A permanent change in antihypertensive therapy upon discharge was documented in 60% (*n* = 15/25) of patients with asymptomatic ICA stenosis and in 75% (*n* = 15/20) of patients with symptomatic ICA stenosis. There was no significant association between a change from pre- to postoperative CBV measurements and neurological symptoms (*p* = 0.08).

A total of 26.7% of patients (*n* = 12/45) suffered from postoperative unilateral headache as symptom suspicious of CHS (patients with asymptomatic ICA stenosis: 32% (*n* = 8/25); patients with symptomatic ICA stenosis: 20% (*n* = 4/20)). Sixty-nine point four percent of ROIs in patients with postoperative unilateral headache showed an increase when comparing pre- and postoperative CBV measurements (patients with symptomatic ICA stenosis: 75%; patients with asymptomatic ICA stenosis: 66.7%). Ten of these 12 patients (83.3%) had preoperative arterial hypertension with the need for antihypertensive medication. In these 10 patients, 65.9% of ROIs showed a postoperative CBV increase. In patients with symptomatic ICA stenosis, 75% of patients with postoperative unilateral headache needed an acute change in antihypertensive therapy and new long-term antihypertensive medication. In patients with symptomatic ICA stenosis, this was the case for 50% of patients with postoperative unilateral headache.

Images of all 12 patients with unilateral headache as symptom suggestive of CHS were evaluated by three different independent observers blinded to the outcomes headache or CHS. For 9 of these 12 patients, a higher subjective CBV measurement after CEA was stated. In two patients, CBV was considered equal before and after CEA, and in one patient, CBV was judged to be lower after CEA. Fleiss’ kappa coefficient for interrater reliability was 0.58.

**Fig. 3:**
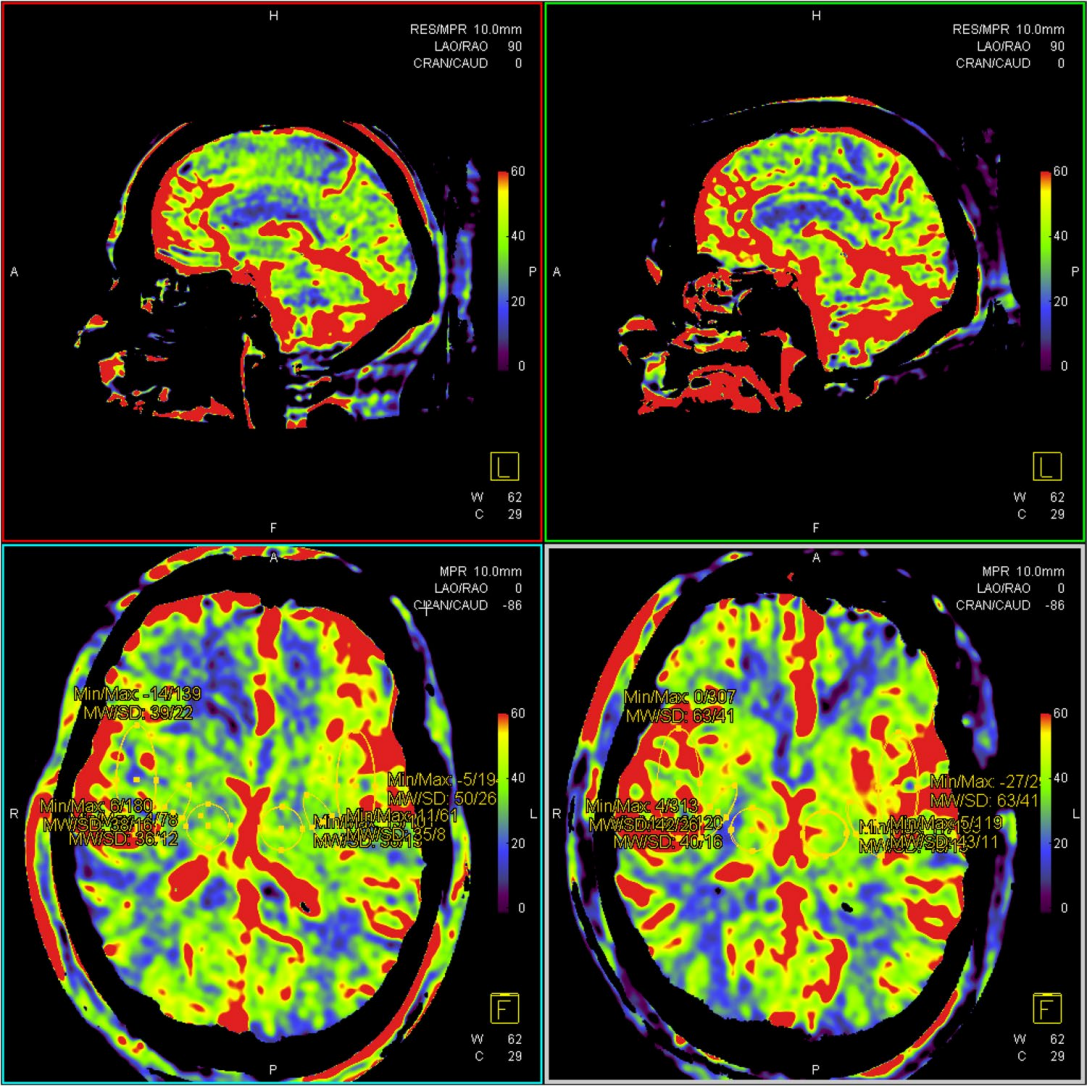
Pre-procedural (left) and post-procedural (right) CBV measurements in ROI. 1: Lateral basal ganglia, 4: thalamus, and 6: internal capsule

**Fig. 4:**
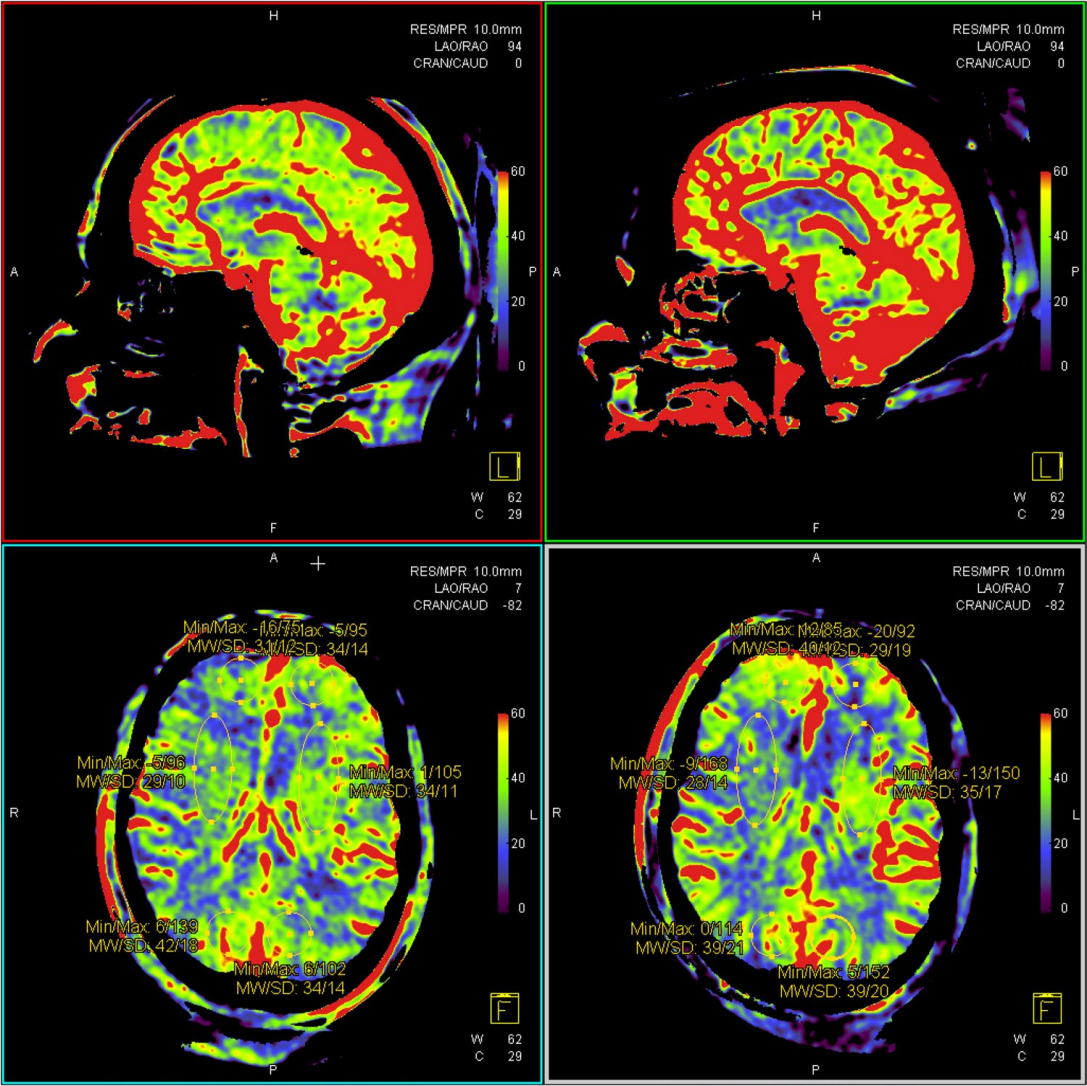
Pre-procedural (left) and post-procedural (right) CBV measurements in ROI. 2: Frontal subcortical white matter, 3: occipital subcortical white matter, and 5: corona radiate

Figures 3 and 4 show ROIs of one of the patients with left-sided symptomatic ICA stenosis who developed postoperative neurological symptoms with unilateral headache as potential symptom of CHS. This patient had acute postoperative hypertension with systolic blood pressure up to 220 mmHg; a permanent change in antihypertensive therapy upon discharge was necessary.

## Discussion

The present study aimed at evaluating the utility of intraoperative CBV measurement for the early detection of CHS in patients undergoing CEA. Although it is often not recognized and its possible sequelae are underestimated in clinical practice with low awareness among physicians, there is evidence that CHS occurs with a relevant incidence following carotid revascularization. Huibers et al. analyzed the incidence of CHS after CAS with a systematic literature review. Thirty-three studies comprising 8731 patients were included. Out of these patients, 341 experienced CHS (4.6% (3.1–6.8%)) [26]. A systematic review and meta-analysis published in 2017 by Galyfos et al. analyzed the incidence of CHS after CEA and CAS. From six studies with 236,537 procedures (216,144 CEA and 18,393 CAS), the authors concluded that CHS was more common in patients after CEA (pooled *OR* = 1.432; *p* = 0.015) [27]. Kirchhoff-Torres et al. stated that CHS is a relevant complication after carotid revascularization, occurring with an incidence of 0.2 to 18.9% in patients undergoing CEA. Because CHS can result in severe complications such as brain oedema and intracerebral or subarachnoid hemorrhage if not treated early and properly, its timely diagnosis is crucial [8].

Lin et al. stated in a review article from 2019 that the “underlying mechanism of CHS is still not fully understood.” One of the current hypotheses on pathophysiology of CHS is described as impairment of cerebral autoregulation. There is no evidence for how long the increase of CBV persists after surgery, but it is described that CHS after endovascular revascularization can occur delayed up to 1 month after the procedure [28].

As mentioned above, baroreceptor dysfunction is another possible hypothesis on pathophysiology of CHS. A breakdown of baroreceptors can lead to the inability to respond to changes in systematic arterial blood pressure. Baroreceptor dysfunction is possible after manipulation of the carotid body such as angioplasty, stentimplantation, or carotid endarterectomy [29].

Two of the strongest putative risk factors for CHS are post-procedural hypertension and recent ipsilateral stroke. The latter assumption does not seem to be supported by the present results. In the study population, CHS was diagnosed in two patients who had asymptomatic stenosis, i.e., no prior ipsilateral neurological event. Postoperative ipsilateral headache suggestive of CHS was also more common in patients with asymptomatic stenosis. The majority of patients with postoperative headache needed postoperative additional antihypertensive medication. Thus, this study was in line with the results from Kirchhoff-Torres et al. that postoperative CHS could correlate with post-procedural hypertension and the need for additional antihypertensive medication [17].

In 2007, Fukuda et al. first evaluated the identification of patients at risk for CHS after CEA by measuring preoperative CBV using perfusion-weighted MR imaging. In their study, a significant correlation between preoperative CBV and increases in cerebral blood flow after CEA was observed. In 47% of patients, immediate hyperperfusion after CEA, defined as cerebral blood flow increase of 100% compared to preoperative values, was seen. Conversely, no patients with normal preoperative CBV exhibited postoperative hyperperfusion. The authors concluded that preoperative CBV measured by perfusion-weighted MR might help to identify patients at risk for cerebral hyperperfusion after CEA [30].

Since Struffert et al. showed in 2011 that CBV measured by flat detector CT was comparable with conventional multislice perfusion CT measurements, intraoperative functional imaging has gained importance. They concluded that the ability to assess cerebral perfusion intraprocedurally might improve the management of ischaemic stroke [31]. In a recent study, Fujimoto et al. investigated the usefulness of intraprocedural CBV measurement by C-arm computed tomography for the evaluation of CHS in patients undergoing CAS. CBV was measured before and immediately after CAS in thirty patients. Postoperative intracerebral hemorrhage was seen in three patients. In those patients, a marked and statistically significant increase was observed in postoperative CBV. The authors concluded that C-arm CT allows CBV measurements immediately after CAS and might enable to assess possible CHS before taking the patient out of the angiography room [18]. There is anecdotal evidence that the intraprocedural detection of cerebral hyperperfusion by flat detector computed tomography is feasible. A case report of a patient undergoing CAS due to symptomatic carotid stenosis showed a dramatically increased CBV in the pertinent hemisphere. The patient did not show neurological changes after strict blood pressure control was established, but a subarachnoid hemorrhage was detected on CT the day after the intervention [32].

The present study was the first to systematically evaluate intraoperative CBV measurement as a tool to identify patients at risk for CHS following CEA. Even though the results failed to reach statistical significance, we observed an increase of CBV in 83.3% of ROIs in patients fulfilling the diagnostic criteria for postoperative CHS. In nine out of the twelve patients with postoperative clinical signs suggestive of CHS, but not diagnosed with CHS by a neurologist, three blinded observers independently stated an increase in CBV.

The study has methodological limitations. First, it was designed as explorative study without a formal power calculation. Therefore, its sample size was relatively small in order to include a sufficient number of patients with a presumably rare event like CHS. This limited its statistical power and probably determined that results were not statistically significant. On the other hand, we did not correct for multiple testing, which increases the likelihood of false positive results. Second, the criteria used to diagnose CHS might be considered somewhat arbitrary. However, there is no widely approved gold standard for its diagnosis. We defined any increase in CBV in a given ROI as hyperperfusion and thus a possible sign of hyperperfusion, which might have led to overdiagnosis in our study. The same holds true for unilateral headache, which might also be induced by non-cerebral causes. Third, the assessment of CBV on images by independent observers was subjective. Yet, this approach might not be too different from the clinical practice of intraoperative CBV assessment. A strength of the study is that it represents the first larger series of patients undergoing CEA with intraoperative CBV measurement with regard to subsequent development of CHS. Furthermore, the quantitative analysis of CBV measurements followed a strict protocol and is therefore valid and reproducible.

## Conclusion

The results of the study suggest that a perioperative increase in CBV might serve as a predictor for the subsequent development of CHS. The findings would need to be corroborated in a larger study. Intraoperative CBV measurements could therefore be used to estimate the risk of CHS and to initiate timely diagnostic and therapeutic measures such as frequent neurological examinations and blood pressure control, and an early initiation of antihypertensive treatment in case of hypertension. Intraoperative CBV measurements are easy to perform in a dedicated setting with trained and experienced staff and a defined protocol and may be considered during CEA depending on availability and the expected risk–benefit ratio for the single patient given the need for intravenous administration of contrast medium and additional radiation exposure.

## References

[1] Eckstein H-H, Kühnl A, Berkefeld J, Dörfler A, Kopp I, Langhoff R et al S3-Leitlinie zur Diagnostik, Therapie und Nachsorge der extracraniellen Carotisstenose; 2. Auflage ─ 03. Februar 2020 AWMF-Registernummer: 004–028

[2] European Carotid Surgery Trialists’ Collaborative Group, Warlow C et al (1998) Randomised trial of endarterectomy for recently symptomatic carotid stenosis: final results of the MRC European Carotid Surgery Trial (ECST). Lancet 351:1379–1387 9593407

[3] Barnett HJ, Taylor DW, Eliasziw M, Fox AJ, Ferguson GG, Haynes RB (1998). Benefit of carotid endarterectomy in patients with symptomatic moderate or severe stenosis. North American Symptomatic Carotid Endarterectomy Trial Collaborators. N Engl J Med..

[4] Halliday A, Mansfield A, Marro J, Peto C, Peto R, Potter J (2004). Prevention of disabling and fatal strokes by successful carotid endarterectomy in patients without recent neurological symptoms: randomized controlled trial. Lancet.

[5] Rothwell PM, Eliasziw M, Gutnikov SA, Warlow CP, Barnett HJ, Carotid Endarterectomy Trialists C (2004) Endarterectomy for symptomatic carotid stenosis in relation to clinical subgroups and timing of surgery. Lancet 363(9413):915–24 10.1016/S0140-6736(04)15785-1 15043958

[6] Halliday A, Harrison M, Hayter E, Kong X, Mansfield A, Marro J (2010). 10-year stroke prevention after successful carotid endarterectomy for asymptomatic stenosis (ACST-1): a multicentre randomized trial. Lancet.

[7] Wieker CM, Harcos K, Ronellenfitsh U, Demirel S, Bruijnen H, Böckler D (2018) Impact of routine completion angiography on outcome following carotid endarterectomy. J Vasc Surg 69(3):824–831 10.1016/j.jvs.2018.06.210 30292609

[8] Walther NKA van Mook, Roger JMW Rennenberg, Geert Willem Schurink, Robert Jan van Oostenbrugge, Werner H Mess, Paul AM Hofman et al (2005) Cerebral hyperperfusion syndrome. Lancet Neurol 4:877–88 10.1016/S1474-4422(05)70251-9 16297845

[9] Sundt TM, Sharbrough FW, Piepgras DG (1981). Correlation of cerebral blood flow and electroencephalographic changes during carotid endarterectomy. Mayo Clin Proc.

[10] Shindo A, Kawai N, Kawakita K (2007). Intracerebral hemorrhage after carotid artery stenting without evidence of hyperperfusion in positron emission tomography. Interv Neuroradiol.

[11] Ogasawara K, Sakai N, Kuroiwa T, Hosada K, Iihara K, Toyoda K (2007). Intracranial hemorrhage associated with cerebral hyperperfusion syndrome following carotid endarterectomy and carotid artery stenting: retrospective review of 4494 patients. J Neurosurg.

[12] Abou-Chebl A, Yadav JS, Reginelli JP, Bajzer C, Bhatt D, Krieger DW (2004). Intracranial hemorrhage and hyperperfusion syndrome following carotid artery stenting: risk factors, prevention, and treatment. J Am Coll Cardiol.

[13] Coutts SB, Hill MD, Hu WY (2003) Hyperperfusion syndrome: toward a stricter definition. Neurosurgery 53(5):1053–1058; discussion 1058–60 10.1227/01.neu.0000088738.80838.74 14580271

[14] Meyers PM, Higashida RT, Phatouros CC, Malek AM, Lempert TE, Dowd CT et al (2000) Cerebral hyperperfusion syndrome after percutaneous transluminal stenting of the craniocervical arteries. Neurosurgery 47(2):335–345; discussion 343–5 10.1097/00006123-200008000-00013 10942006

[15] Mori T, Fukuoka M, Kazita K, Mima T, Mori K (1999). Intraventricular hemorrhage after carotid stenting. J Endovasc Surg.

[16] Wu TY, Anderson NE, Barber PA (2012). Neurological complications of carotid revascularization. J Neurol Neurosurg Psychiatry.

[17] Kirchoff-Torres KF, Bakradze E (2018). Cerebral hyperperfusion syndrome after carotid revascularization and acute ischemic stroke. Curr Pain Headache Rep.

[18] Fujimoto M, Itokawa H, Moriya M, Okamoto N, Sasanuma J (2018). Evaluation of cerebral hyperperfusion after carotid artery stenting using C-Arm CT measurements of cerebral blood volume. Clin Neuroradiol.

[19] Piepgras DG, Morgan MK, Sundt TM, Yanagihara T, Mussman LM (1988). Intracerebral hemorrhage after carotid endarterectomy. J Neurosurg.

[20] Pomposelli FB, Lamparello PJ, Riles TS, Craighead CC, Giangola G, Imparato AM (1988). Intracranial hemorrhage after carotid endarterectomy. J Vasc Surg.

[21] Ouriel K, Shortell CK, Illig KA, Greenberg RK, Green RM (1999) Intracerebral hemorrhage after carotid endarterectomy: incidence, contribution to neurologic morbidity, and predictive factors. J Vasc Surg 29(1):82–7; discussion 87–9 10.1016/s0741-5214(99)70362-9 9882792

[22] Bley T, Strother CM, Pulfer K, Royalty K, Zellerhoff M, Deuerling-Zheng Y (2010). C-arm CT measurement of cerebral blood volume in ischemic stroke: an experimental study in canines. AJNR Am J Neuroradiol.

[23] Struffert T, Deuerling-Zheng Y, Kloska S, Engelhorn T, Strother CM, Kalender WA (2010). Flat detector CT in the evaluation of brain parenchyma, intracranial vasculature, and cerebral blood volume: a pilot study in patients with acute symptoms of cerebral ischemia. AJNR Am J Neuroradiol.

[24] Willams B, Mancia G, Spiering W, Agabiti Rosei E, Azizi M, Burnier M et al (2018) ESC/ESH guidelines for the management of arterial hypertension. Eur Heart J 39(33):3021–3104. 10.1093/eurheartj/ehy339 10.1093/eurheartj/ehy339 30165516

[25] Landis JR, Koch GG (1977). The measurement of observer agreement for categorical data. Biometrics.

[26] Huibers AE (2018). Cerebral hyperfusion syndrome after carotid artery stenting: a systematic review and meta-analysis. Eur J Vasc Endovasc Surg.

[27] Galyfos G, Sianou A, Filis K (2017). Cerebral hyperfusion syndrome and intracranial hemorrhage after carotid endarterectomy or carotid stenting: a meta-analysis. J Neurol Sci.

[28] Lin Y-H, Liu H-M (2020). Update on cerebral hyperperfusion syndrome. J NeuroIntervent Surg.

[29] Nouraei SAR, Al-Rawi PG, Sigaudo-Roussel D, Giussani DA, Gaunt ME (2005). Carotid endarterectomy impairs blood pressure homeostasis by reducing the physiologic baroreflex reserve. J Vasc Surg.

[30] Fukuda T, Ogasawara K, Kobayashi M, Komoribayashi N, Endo H, Inoue T (2007). Prediction of cerebral hyperperfusion after carotid endarterectomy using cerebral blood volume measured by perfusion-weighted MR imaging compared with single-photon emission CT. AJNR Am J Neuroradiol.

[31] Struffert T, Deuerling-Zheng Y, Kloska S, Engelhorn T, Boese J, Zellerhoff M (2011). Cerebral blood volume imaging by flat detector computed tomography in comparison to conventional multislice perfusion CT. Eur Radiol.

[32] Terada Y, Hatano T, Nagai Y, Hayase M, Oda M, Nakamura T (2014) Intraprocedural detection of cerebral hyperperfusion by flat detector computed tomography in the evaluation of cerebral blood volume during carotid artery stenting. Interv Neuroradiol 20(4):502–9 10.15274/INR-2014-10044 PMC4187447 25207915

